# Canine mammary cancer cells direct macrophages toward an intermediate activation state between M1/M2

**DOI:** 10.1186/s12917-015-0473-y

**Published:** 2015-07-15

**Authors:** Breno C.B. Beirão, Teresa Raposo, Lisa Y. Pang, David J. Argyle

**Affiliations:** The Royal (Dick) School of Veterinary Studies and Roslin Institute, The University of Edinburgh, Easter Bush, Midlothian, Scotland EH25 9RG UK; Department of Veterinary Sciences, Universidade de Trás-os-Montes e Alto Douro, 5001-801 Vila Real, Portugal

**Keywords:** Tumour-associated macrophages, Dog, Mammary, CSF-1, CCL2, Twist-1

## Abstract

**Background:**

Canine mammary carcinoma is the most common cancer in female dogs and is often fatal due to the development of distance metastasis. The microenvironment of a tumour often contains abundant infiltrates of macrophages called tumour-associated macrophages (TAMs). TAMs express an activated phenotype, termed M2, which sustains proliferation of cancer cells, and has been correlated with poor clinical outcomes in human cancer patients. Cancer cells themselves have been implicated in stimulating the conversion of macrophages to a TAM with an M2 phenotype. This process has yet to be fully elucidated. Here we investigate the interplay between cancer cells and macrophages in the context of canine mammary carcinoma.

**Results:**

We show that cancer cells inhibit lipopolysaccharide (LPS)-induced macrophage activation. Further, we show that macrophage associated proteins, colony-stimulating factor (CSF)-1 and C-C motif ligand (CCL)-2, stimulate macrophages and are responsible for the effects of cancer cells on macrophages. We suggest the existence of a feedback loop between macrophages and cancer cells; while cancer cells influence the phenotype of the TAMs through CSF-1 and CCL2, the macrophages induce canine mammary cancer cells to upregulate their own expression of the receptors for CSF-1 and CCL2 and increase the cancer cellular metabolic activity. However, these cytokines in isolation induce a phenotypic state in macrophages that is between M1 and M2 phenotypes.

**Conclusions:**

Overall, our results demonstrate the extent to which canine mammary carcinoma cells influence the macrophage phenotype and the relevance of a feedback loop between these cells, involving CSF-1 and CCL2 as important mediators.

**Electronic supplementary material:**

The online version of this article (doi:10.1186/s12917-015-0473-y) contains supplementary material, which is available to authorized users.

## Background

Mammary tumours are the most common neoplasms that affect female dogs (*Canis familiaris*), constituting half of all tumours in female dogs. From these approximately half are considered malignant [2, 3, 9]. In both women and dogs, the incidence of mammary tumour increases with age, rarely occurring before 25 and 5 years of age, respectively [23] and is hormone dependent [19]. Canine mammary carcinomas have epidemiologic, clinical, morphologic and prognostic features similar to those of human breast cancer and therefore represent a comparative model to understand the underlying molecular mechanisms of carcinogenesis and impacts of the tumour microenvironment in both species [18, 19, 23].

Tumour-associated macrophages (TAMs) are major orchestrators of the tumour microenvironment by: sustaining the proliferation of both cancer and stromal cells; facilitating angiogenesis; suppressing the anti-tumoural functions of T-lymphocytes; and enhancing the survival of cancer stem cells [1–3, 9, 18, 19, 23]. It has subsequently been shown that macrophage depletion improves outcome in several cancer models [1, 17, 33] and validates TAMs as potential therapeutic targets.

Macrophages are immune cells found in essentially all tissues of the body, where they engulf apoptotic cells and pathogens and produce immune effector molecules. These cells are remarkably plastic and can change their functional phenotype depending on the environmental cues they receive: macrophages can increase inflammation and stimulate the immune system, and in contrast, also play an important anti-inflammatory role and decrease immune reactions through the release of cytokines. Macrophages that are pro-inflammation are called M1 macrophages, whereas those that decrease inflammation and encourage tissue repair are called M2 macrophages. TAMs have an M2 phenotype and are predominately activated by IL-4, IL-10 and CSF-1 secreted by the tumour, whereas M1 macrophages are classically activated by lipopolysaccharide (LPS) and IFNγ and driven to phagocytosis [19]. Recent work has highlighted the plasticity of macrophages and indicates that transition forms of M1/M2 macrophages coexist during tumour progression [27]. For example, an intermediary form of macrophages has recently been identified as regulatory macrophages, presenting elevated IL-10 and low IL-12 secretion [4]. During the M1-to-M2 transition of macrophages the molecular changes occurring can have profound effect on breast cancer cell behaviour; notably, the levels of the cytokines CSF-1 and CCL2 are elevated during this transition [16, 32]. CSF-1 and CCL2 are known modulators of the fate of macrophages, both driving the production of M2 cells and being correlated to poor prognosis in cancer [5, 28].

Our understanding of the interaction between TAMs and cancer cells is still evolving [6], as is the very classification of M1/M2 macrophages [20]. Much is still debated with regards to the origin, the variety of phenotypes, and the actual roles of macrophages within tumours. While the predominating view is that the tumour provides an environment that promotes an M2 phenotype, consensus is shifting towards the idea that each individual macrophage is a distinct unit that differs from its counterparts [20, 21]. For example, there is evidence that while capable of inducing tumour invasion and expressing M2 markers, TAMs also retain the ability to kill cancer cells when mediated by anti-cancer antibodies [12].

In this study we aim to further understand the mutual effects between cancer cells and macrophages by investigating the role of CSF-1 and CCL2 stimulation on this interaction and promotion of an M2 phenotype within the context of canine mammary carcinoma. We found that while CSF-1 and CCL2 drive the onset of M2 characteristics, we propose that these cytokines also mediate activation of macrophages to a state that is not fully polarized.

## Results

### Macrophage activation can be inhibited by mammary carcinoma cells

**Fig. 1:**
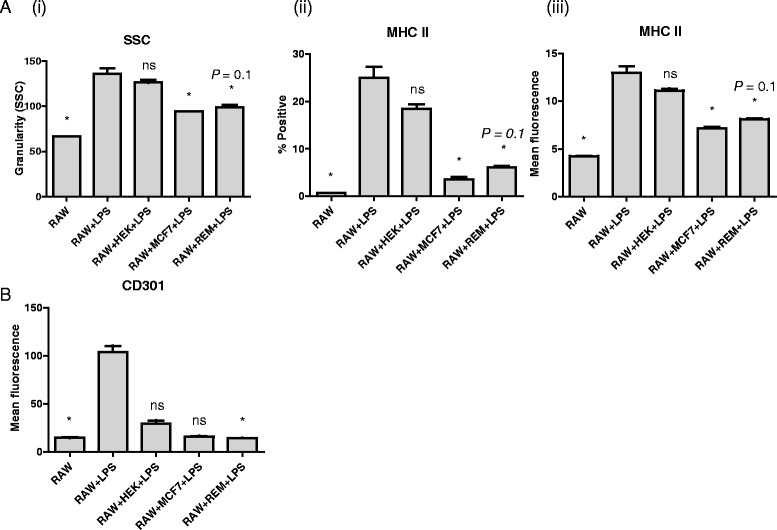
Cancer cells inhibit LPS induced activation of macrophages. Macrophages (RAW) were co-cultured with either canine mammary carcinoma (REM), Human mammary cancer cells (MCF7) or non-cancerous cells (HEK293T), stimulated with 1 μg/ml LPS for 48 h. LPS-induced activation of macrophages was measured by changes in granularity (**a** (i)), percentage of MHC II+ cells (**a** (ii)) or mean fluorescence intensity of MHC II (**a** (iii)), and expression of the M2 marker, CD301 (**b**). Macrophages were distinguished from cancer cells in flow cytometry by CSFE staining. All experiments are replicates of 4. Asterisks indicate statistically significant differences in relation to LPS-treated cells, where *P* < 0.05 (unless where indicated) by Kruskal-Wallis test; “ns” indicates non-significant differences

Macrophages are activated by LPS, a major component of the outer membrane of gram-negative bacteria. Here, LPS-induced macrophage activation was measured by increased expression of MHC II and enhanced granularity, as demonstrated by an increase in side scatter (SSC) on flow cytometry. The enhanced side scatter may indicate accumulation of enzyme-containing granules and cellular activation [24]. To determine the interplay between cancer cells and macrophages, we co-cultured a macrophage cell line (RAW) with either canine mammary carcinoma cells (REM134), human mammary carcinoma cells (MCF-7) or non-cancerous control cells (HEK293T). The presence of either REM134 or MCF-7 cells significantly reduced LPS-induced activation of RAW macrophages as measured by both granularity (Fig. 1a(i)) and expression of MHC II (Fig. 1a(ii) and (iii)). The presence of non-cancerous HEK293T cells had only a minor effect on macrophage activation (Fig. 1a, b). Co-culture of macrophages and REM134 cells was also able to inhibit the expression of M2 macrophage marker CD301 in response to LPS-induced activation, contrary to what would be expected; this cell surface marker was anticipated to be increased with cancer cell-stimulation of the macrophages (Fig. 1b). These results demonstrate that both canine and human mammary cancer cells can condition macrophages into a reduced activation state in the presence of LPS, characteristic of M2 macrophages, but independent of the M2 marker CD301.

### LPS-induced activation of macrophages is CCR2 dependent

**Fig. 2:**
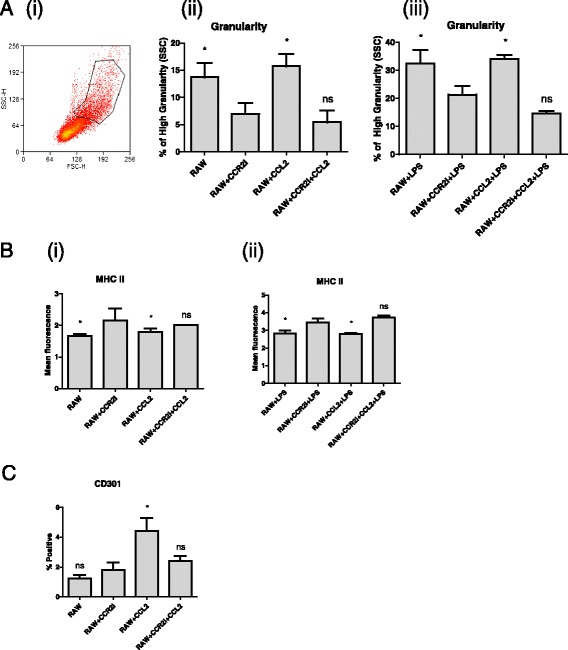
M1/M2 polarization of macrophages involves CCR2 signalling. RAW cells were pre-treated for 1 h with 10 μM RS102895 hydrochloride, a small molecule inhibitor of CCR2 (CCR2i) prior to stimulation with 1 μg/ml LPS for 48 h. An equivalent volume of DMSO was used as a vehicle control. LPS-induced activation of macrophages was measured by changes in the percentage of high granularity cells (**a** (i)) before (**a** (ii)) and after LPS-treatment (**a** (iii)), expression of MHC II before (**b** (i)) or after LPS stimulation (**b** (ii)), and expression of the M2 marker, CD301 (**c**). All experiments are replicates of 3. Asterisks indicate statistically significant differences in relation to the CCR2i treated cells, where *P* < 0.05 by Kruskal-Wallis (Figure A) or ANOVA (Figures B and C); “ns” indicates non-significant differences

The cytokine CCL2 is important for macrophage recruitment and driving the M1 to M2 transition [29]. Here, we utilised the specific small molecule inhibitor of the CCL2 receptor (CCR2). Macrophages were pre-incubated with either the inhibitor alone or the inhibitor and exogenous CCL2 prior to treatment with LPS. We found that inhibition of CCR2 had dual effects: significantly inhibiting the activation of macrophages as measured by the amount of high granularity cells (Fig. 2a), and simultaneously increasing the expression of MHC II before (Fig. 2b (i)) and after (Fig. 2b (ii)) LPS-stimulation. The high granularity cells were separated by FACS and were confirmed not to be doublets (not shown). Interestingly, the basal expression level of CD301 was induced by CCL2 but compared to control cells was unaffected by the presence of the CCR2 inhibitor (Fig. 2c). Therefore, while MHC II and CD301 expression levels indicate that CCL2 signalling leads to M2 characteristics, the cellular granularity indicates that inhibiting CCR2 actually can decrease this parameter of activation. Overall, these results indicate that CCR2 signalling is important for the formation of alternatively activated macrophages, but blocking CCR2 also decreases the population of naturally highly granular cells within the macrophage cell line.

### Effects of mammary carcinoma cells on macrophages is CSF1 dependent

**Fig. 3:**
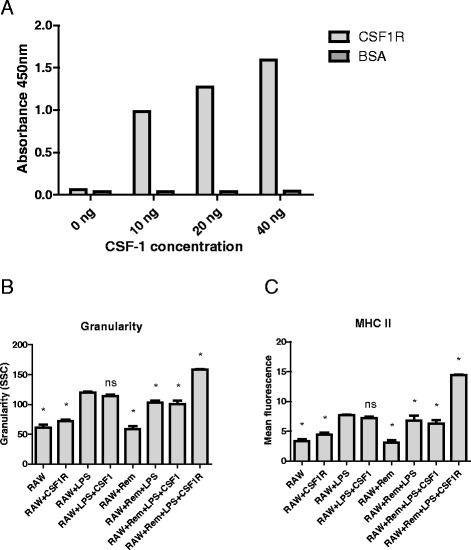
Cancer cell effects on macrophages are CSF-1 dependent. Canine recombinant CSF-1R binds to CSF-1 as detected by ELISA. BSA was used as a control protein for coating the plate (**a**). REM134 cells, RAW cells and soluble canine recombinant CSF1R (1 μg/ml every 24 h) were co-cultured for 3 days prior to LPS-induced activation of macrophages. Activation of macrophages was measured by granularity (**b**) and expression of MHC II (**c**). All experiments are replicates of 3. Asterisks indicate statistically significant differences in relation to the LPS treated cells, where *P* < 0.05 by ANOVA; “ns” indicates non-significant differences

Colony stimulating factor-1 (CSF-1) is associated with M2 activation of macrophages in cancer [29]. To investigate the role of CSF-1 in the interaction between cancer cells and macrophages, we utilised soluble recombinant canine CSF-1 receptor (rcCSF-1R) to competitively inhibit free CSF-1 [30] in co-culture experiments with RAW cells and REM134 cells. The ability of the soluble recombinant receptor to bind CSF-1 was determined by ELISA (Fig. 3a). The presence of canine mammary carcinoma cells decreased the LPS-induced activation of macrophages as determined by granularity (Fig. 3b) and expression of MHCII (Fig. 3c). This effect was rescued and enhanced by inhibition of CSF-1, whereby LPS-induced activation of macrophages – as determined by granularity (Fig. 3b) and expression of MHCII (Fig. 3c) – was elevated 2.11-fold and 1.53-fold respectively. This implies that cancer cell mediated effects on macrophages are at least partially dependent on CSF-1.

### Effects of macrophages on mammary carcinoma cells involves CSF1 and CCL2

**Fig. 4:**
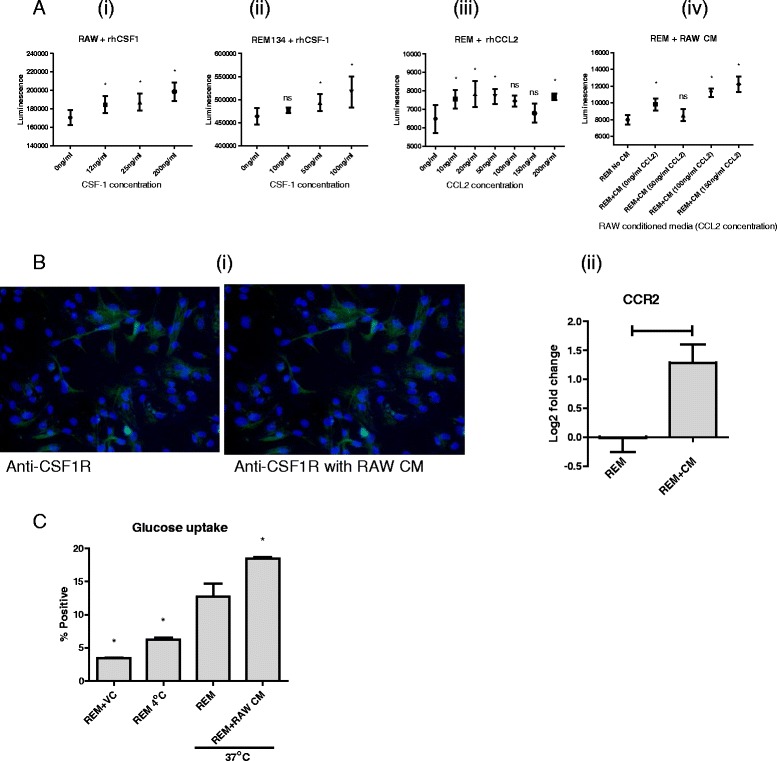
CSF-1 and CCL2 contribute to cancer cell proliferation and activity. Macrophages (**a** (i)) and REM134 (**a** (ii)) cells were incubated with increasing doses of CSF-1. Cellular proliferation was measured after 48 h. PBS + 0.1 % BSA was used a vehicle control at 0 ng/ml. REM134 cells were incubated with increasing concentrations of CCL2 (**a** (iii)) or conditioned media (CM) from CCL2 treated macrophages (**a** (iv)). Cellular proliferation was measured after 48 h. PBS + 0.1 % BSA was used a vehicle control at 0 ng/ml. Experiments are replicates of 6. Statistical analysis considered each group against the control group. Expression of CSF-1R was assayed in Lilly cancer cells, after exposure to macrophage conditioned media, by immunofluorescence (**b** (i)). Expression of CCR2 was measured in REM134 cells, after exposure to macrophage-conditioned media, by qRT-PCR (**b** (ii)). Cellular activity was measured by glucose uptake. REM134 cells were pre-incubated with macrophage-conditioned media for 72 h prior to analysis. Ethanol was used as a vehicle control (VC). Cells were incubated at 4 °C as a negative control (**c**). Figures B and C are replicates of 3. Statistical analysis considered each group against the REM group. Asterisks or horizontal bars indicate statistically significant differences to the 0 ng/ml or REM group, where *P* < 0.05 by Kruskal-Wallis (Figure A) or Mann–Whitney (Figures B and C); “ns” indicates non-significant differences

We have shown that CCL2 is required for driving LPS-induced activation of macrophages, and that CSF-1, secreted from cancer cells, induces polarisation of macrophages. We next assessed the effect of these cytokines on canine cancer cells. Both macrophages (Fig. 4a (i)) and REM134 (Fig. 4a (ii)) cells were incubated independently with increasing concentrations of CSF-1, and showed a dose-dependent increase in cell proliferation. Interestingly, REM134 cells incubated with increasing doses of CCL2 showed marginal effects on cell proliferation – 1.2-fold increase in proliferation at the highest doses used – which was not dose-dependent at the stated doses ((Fig. 4a (iii)). As an alternative method to assess the effect of CCL2 on REM134 cells, we cultured macrophages with increasing doses of CCL2 and then harvested the media, termed macrophage conditioned media. This was subsequently used to treat REM134 cells, and induced a CCL2 dose-dependent increase in cell proliferation (Fig. 4a (iv)). This data suggests that cancer cell proliferation can be stimulated in a dose-dependent manner directly by CSF-1 and indirectly via CCL2. Furthermore, addition of macrophage conditioned media to mammary cancer cells seemed to induce the expression of the receptors required for the signalling of CSF-1 (CSF-1R) (Fig. 4b (i)) and CCL2 (CCR2) (Fig. 4b (ii)).

We also analysed the cellular activity of REM134 cells after treatment with macrophage-conditioned media by assaying for glucose uptake. Here REM134 cells treated with macrophage-conditioned media showed a higher level of glucose uptake compared to untreated cells (Fig. 4c). Glucose uptake of REM134 cells was also measured at 4 °C as a negative control and with the addition of ethanol as a vehicle control (VC). These results indicate that cancer cells respond to factors secreted by macrophages by upregulating: expression of key signalling receptors; cellular proliferation; and cellular activity.

### Proliferation of canine mammary carcinoma cell lines is dependent on CSF-1R

**Fig. 5:**
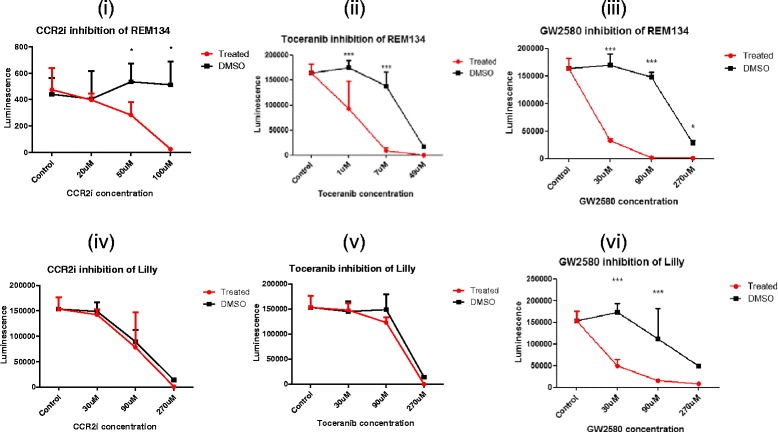
Proliferation of canine mammary carcinoma cells is CSF-1R dependent. Canine mammary carcinoma cell lines, REM134 and Lilly were treated with increasing doses of either CCR2 small-molecule inhibitor (i-iv), toceranib (ii-v), or CSF-1R inhibitor  (iii-vi) for 48 h before assaying for cell viability. All experiments are replicates of 3. Asterisks indicate statistically significant differences to the DMSO group, where *P* < 0.05 by ANOVA

To determine the effect of inhibition of CSF-1 and CCL2 signalling on cancer cells we utilised the small molecule inhibitors of CSF-1R (GW2580) and CCR2 (RS102895). As a control we used the tyrosine kinase inhibitor, toceranib. Toceranib (Palladia®) inhibits phosphorylation of c-kit receptor tyrosine kinase and cell proliferation of REM134 cells in a dose dependent manner (*unpublished data*). Here we show that REM134 cell proliferation is inhibited in a dose dependent manner by inhibition of CCR2 (Fig. 5(i)), cKit (Fig. 5(ii)), and CSF-1R (Fig. 5(iii)). To determine if these effects are consistent in canine mammary carcinoma cell lines, we utilised the inflammatory mammary carcinoma cell line, Lilly (a kind gift from Dr R. de Maria, University of Turin, Italy). Lilly cells showed a dose-dependent decrease in proliferation when treated with GW2580 (Fig. 5(vi)), but not with the inhibitor of CCR2 ((Fig. 5(iv)), or toceranib (Fig. 5(v)), highlighting cell line dependent responses to treatment. However these results show that the proliferation potential of both canine mammary carcinoma cell lines tested are dependent on CSF-1R signalling.

### Dual role of CSF-1 in stimulation of macrophages

**Fig. 6:**
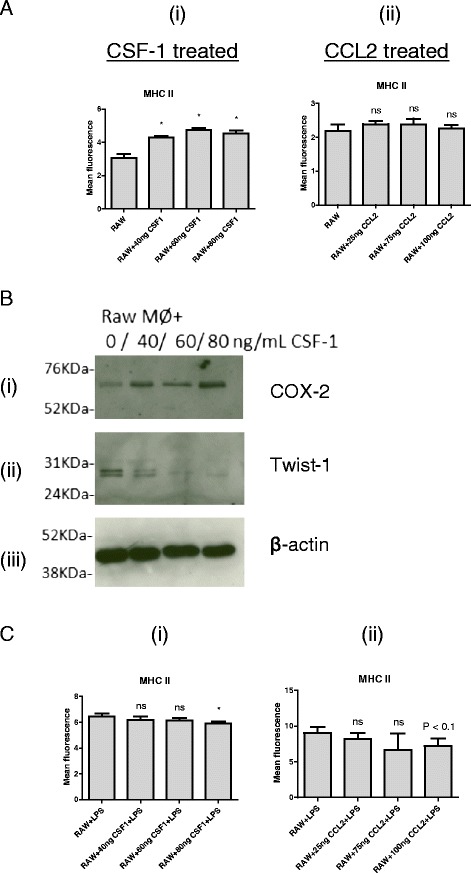
The dual role of CSF-1 in macrophage activation. Macrophages were incubated with media containing increasing concentrations of either recombinant CSF-1 (**a** (i)) or recombinant CCL2 (**a** (ii)) for 2 days prior to analysis of MHC II expression by flow cytometry. Protein expression levels of COX-2 and Twist were determined by Western blotting. β-actin was used as a loading control. 20 μg protein loaded (**b**). Macrophages were incubated with media containing increasing concentrations of either recombinant CSF-1 (**c** (i)) or recombinant CCL2 (**c** (ii)) for 2 days prior LPS-induced activation (300 μg/ml LPS for 48 h). MHC II expression levels were determined by flow cytometry. Figures (i) are replicates of 6, Figures (ii) are replicates of 3. Asterisks indicate statistically significant differences to the RAW or RAW + LPS group, where *P* < 0.05 by Kruskal-Wallis (Figures A(i) and C(ii)) or ANOVA, unless where indicated; “ns” indicates non-significant differences

CSF-1 is an important mediator of the interaction between macrophages and cancer cells. Here we investigate the effect of recombinant CSF-1 on macrophages. Macrophages were grown in media containing increasing amounts of recombinant CSF-1 for 48 h and then analysed for expression of the M1 marker, MHC II. Basal MHC II expression levels increased in a dose dependent manner in response to recombinant CSF-1 (Fig. 6a (i)). In contrast, exposing macrophages to media containing increasing concentrations of CCL2 had no significant effect on basal MHC II levels (Fig. 6a (ii)). To understand the effect of CSF-1 in driving pro or anti-inflammatory properties, we studied two markers of these characteristics by Western blot. Exposure of macrophages to recombinant CSF-1 increased the expression of the inflammation marker, COX-2 and decreased expression of the anti-inflammatory marker, Twist-1 (Fig. 6b), indicating that CSF-1 conditioning of macrophages leads to an inflammatory phenotype.

LPS-induced activation of macrophages in the indicated media showed that both CSF-1 (Fig. 6c (i)), and CCL2 (Fig. 6c (ii)) mediate a dose-dependent decrease in MHC II levels in response to LPS activation, indicating that CSF-1 plays a dual role in macrophage activation.

### Reduced expression of Twist-1 mimics the effect of CSF-1 stimulation

**Fig. 7:**
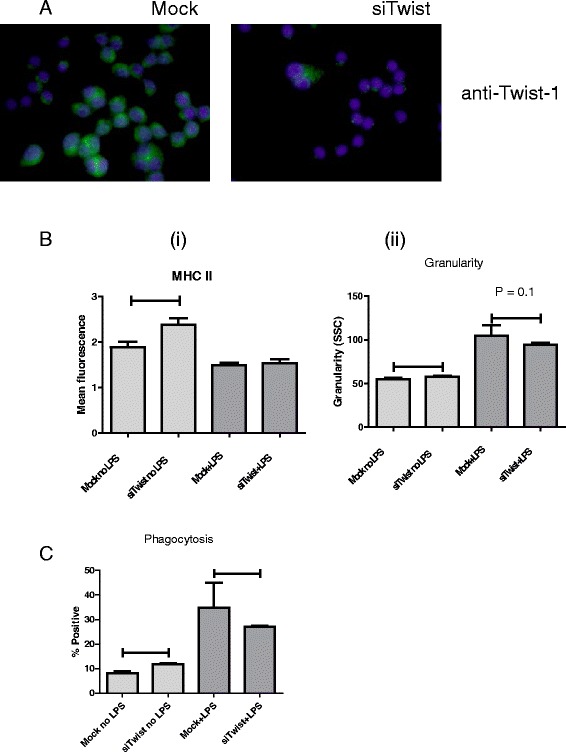
CSF-1 and Twist-1 are key mediators of macrophage activation. Twist-1 protein levels were depleted by treating with siRNA against Twist-1 for 24 h. Immunofluorescence was carried out using anti-Twist-1 antibody (**a**). The effects of the reduction of Twist-1 levels on LPS-induced activation of macrophages were assayed by expression of MHC II (**b** (i)); granularity (**b** (ii)); and phagocytosis (**c**). All experiments are replicates of 3. Horizontal bars indicate statistically significant differences on a Student t-test, where *P* < 0.05, unless where indicated; “ns” indicates non-significant differences

To determine if the effect of CSF-1 on macrophages is mediated via the transcription factor Twist-1, we reduced the levels of Twist-1 protein in macrophages using siRNA to Twist-1 (Fig. 7a). This resulted in a significant increase in basal MHC II levels compared to mock transfected cells (Fig. 7b (i)). However LPS-activation of macrophages showed no change in MHC II levels and this was unaffected by Twist-1 expression levels. Granularity was again used as a measure of macrophage activation. Reduction of Twist-1 levels increased basal granularity marginally but with statistical significance, and significantly decreased LPS-induced activation of macrophages (Fig. 7b (ii)).As a measure of cellular function we assayed the effect of Twist-1 knock down on phagocytosis, and identified a similar pattern to granularity. Reduction of Twist-1 levels slightly increased basal levels of phagocytosis compared to control, but upon activation of macrophages by LPS there was significantly decreased phagocytosis (Fig. 7c). These results indicate that reduction in Twist-1 levels mimics the effects of treating macrophages with recombinant CSF-1 and suggests that CSF-1 acts upstream of Twist-1 to mediate macrophage polarisation.

## Discussion

Distinct macrophage subsets have been linked with either protective or pathogenic roles in cancer. A protective role has been described for M1 macrophages, which activate tumour-killing mechanisms and antagonise the suppressive activity of TAMs. In contrast, TAMs suppress adaptive tumour-specific immune responses and promote tumour growth, invasion, metastasis, stroma remodelling and angiogenesis. TAMs have a suppressive M2-like phenotype. Accumulating evidence from many tumour models suggests that macrophages contribute to tumour progression, with increasing numbers of TAMs correlating with poor outcomes. Here we investigated the interplay between canine mammary carcinoma cells and macrophages. We have shown that cancer cells and macrophages have mutual effects on each other, and that the cytokines CSF-1 and CCL2 are important mediators of this interaction. Furthermore, we have identified that CSF-1 is likely to signal via the transcription factor Twist-1 to exert its effects on macrophage activation.

Our data corroborates previous studies. Wasserman *et al.* (2012) demonstrated the capacity of several canine cancer cells to inhibit macrophage MHC II expression, therefore driving TAMs into the alternative M2-activation pathway [32]. Król et al. (2012) [16] showed that LPS-induced activation of macrophages was inhibited by co-culturing macrophages with canine mammary cancer cells. Here we utilised canine mammary carcinoma cells and showed by cellular granularity and MHC II expression of macrophages that REM134 cells can inhibit LPS-induced activation of macrophages.

There is currently controversy regarding the dogma of classic and alternative macrophage activation, termed M1 and M2, respectively. As research into macrophage biology has evolved, so has the growing amount of information regarding recognition receptors, cytokines, and the signalling and genetic programs behind them that control an increasing number of functions of macrophages. Therefore there is a need to recognise a broader functional repertoire of macrophages that may not fit into the distinct M1 and M2 classifications [20]. Furthermore, tumour microenvironments, compared to a healthy tissue, are haphazard and may contain areas of hypoxia, higher lactate, extracellular acidosis and glucose starvation [8]. Within a tumour multiple and different M1 and M2 stimuli may act on macrophages, and in this context, macrophages may not form distinct activation subsets nor clonally expand, leading to a spectrum of macrophage phenotypes. As a marker of M2-activation we used CD301. In contrast to the granularity and MHC II expression data, expression of CD301 increased after LPS-activation but was unaffected by the presence of cancer cells, indicating that, in these culture conditions, macrophages under the influence of cancer cells are in an activation state between the extremes of the M1 or M2 spectrum.

CSF-1 and CCL2 have well-characterised roles in macrophage activation including inducing macrophage survival and recruitment [17, 29]. Expression of both CSF-1 and CCL2 have been independently correlated with cancer progression in several tumour types [28]. Our studies show that blocking the receptor of CCL2, CCR2, with a small molecule inhibitor could increase macrophage activation, and this supports previous studies where RAW264.7 macrophages are able to produce this cytokine [22] and, by blocking this autocrine signalling, induce cellular activation. This may be mediated through Activin A, which can simultaneously alter the expression of CCR2 and CCL2 in macrophages, depending on their previous activation state [29]. Blocking CCR2 signalling is expected to induce the expression of Activin A, which is known to drive MHC II expression in macrophages, as well as phagocytosis and other M1 characteristics [11]. We showed that in the presence of LPS the effects of blocking CCR2 were more pronounced. Here, blocking CCR2 in macrophages allowed for a marked increase in cellular activation. The addition of rhCCL2 could not reverse this effect, demonstrating that blockade of the receptor was complete. Interestingly, it has been shown that LPS treatment alone can induce CCL2 expression in RAW264.7 macrophages as a negative feedback, since its expression is controlled by STAT-3, an immune regulator [22, 31]. The effect of CCL2 on macrophages was confirmed by CD301 expression. Adding recombinant CCL2 increased expression of this marker. Overall, the results with CCL2 indicate a shift towards an M2 activation phenotype in the presence of CCL2/CCR2 signalling. Another work found that co-culture of macrophages and canine cancer cells reduced the relative expression of CCL2 by the macrophages, but at the same time induced its expression by cancer cells [16], therefore supporting the hypothesis that macrophages will be directed towards an M2 phenotype in the presence of cancer cells. However, CCR2 blockade also had the opposite effect of reducing the percentage of highly-granular macrophages. These highly-granular cells represent only a small fraction of the total macrophage population, but are likely to represent an activated subset of the cellular population [24]. Therefore, the reduction in the highly-granular subgroup of macrophages in the absence of CCR2 signalling points away from the more accepted role of CCR2 inducing M2 polarization of TAMs.

As with CCL2, CSF-1 had a negative impact on LPS-induced macrophage activation. While adding recombinant CSF-1 reduced macrophage expression of MHC II following LPS activation, blocking it with a soluble recombinant CSF-1R allowed a marked activation of these cells. This indicates that by blocking CSF-1R signalling, an inflammatory phenotype can be obtained, even in the presence of cancer cells.

**Fig. 8:**
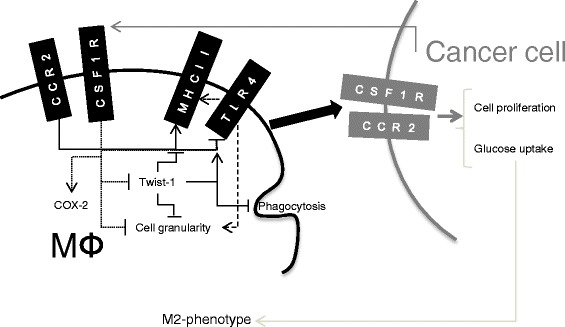
Schematic representation of the molecular interactions following macrophage and cancer cell activation. Arrows indicate that the pathway is increased; blunt-ended lines represent a negative effect on another receptor/pathway

Both macrophages and canine mammary cancer cells are able to proliferate in the presence of rhCSF-1. While CCL2 itself induces some cancer cell proliferation, CCL2 conditioning of the macrophages increases the effect that these cells have on cancer cell proliferation. Macrophage conditioned media is also capable of inducing expression of CSF-1R and CCR2 by mammary carcinoma cells, indicating that there is a cycle whereby cancer cells induce macrophage proliferation and phenotypic change, and the presence of macrophages leads to cancer cell growth, with both processes occurring, at least partially, through CSF-1R/CCR2 signalling (Fig. 8). CSF-1R expressed by cancer cells does not have any mutations compared to the native receptor (data not shown), indicating that it must depend on either autocrine or paracrine signalling to support cancer expansion. CSF-1R has been associated with mammary cancer aggressiveness in dogs [14, 15], and the expression of the receptor has been shown to be increased in canine mammary adenocarcinoma cells in co-cultures with macrophages [16].

The effect of macrophage conditioned media on the cancer cells is biologically relevant since it increases cancer cell metabolism, as measured by cellular glucose uptake and cellular proliferation. Increased expression of CCL2 can induce insulin resistance [13], which is usually associated with hyperinsulinemia [25] and increased glucose uptake by cancer cells. Lactic acid production by tumour cells, generated as a consequence of glucose metabolism, has been shown to be central for the signalling that induces M2-macrophages (as measured by Arg-1 expression) and vascular endothelial growth factor expression [6]. Therefore, when in contact with macrophages, cancer cells will consume more glucose, thereby reinforcing an M2 phenotypic state on the macrophages.

CSF-1R signalling is relevant both for macrophages and cancer cells. However the role of CCL2 produced by the cancer cells, as described previously [16], appears to be confined to altering the macrophage phenotype, as cancer cells themselves were unaffected by inhibition of CCR2. The proliferative response of cancer cells to CCL2-treated macrophage CM is probably due to activation of other receptors in the cancer cells by soluble factors secreted from macrophages. This highlights the complexity of the tumour microenvironment and the importance of the secretome in the interplay between cancer cells and TAMs.

Twist-1 is a transcription factor that has been associated with macrophage activation and function and in the interaction between tyrosine kinase receptors (such as CSF-1R) and Toll-like receptors (such as TLR4, the receptor for LPS) [7]. To assess the importance of Twist-1 signalling in the M1-to-M2 transition, expression of Twist-1 was depleted in macrophages. A decrease in Twist-1 protein levels lead to increased macrophage activation, as measured by phagocytosis capacity, cell granularity and MHC II expression. However, when LPS was added, Twist-1 knock down only partially impeded macrophage activation by LPS. Therefore, depletion of Twist-1 had similar effects to treating macrophages with recombinant CSF-1, indicating that CSF-1 may signal through Twist-1 to mediate the M1-to-M2 transition.

## Conclusions

In conclusion we have characterised the interplay between cancer cells and macrophages. We have shown that there is a cycle mediated by the cytokines CSF-1 and CCL2, whereby cancer cells induce macrophage proliferation and phenotypic changes, and macrophages stimulate cancer cell proliferation. We have also highlighted the importance of the transcription factor Twist-1 in macrophage activation and that cancer-related changes in glucose metabolism reinforce the M2 phenotypic state of macrophages. The tumour microenvironment is highly complex and we have shown dual roles for the cytokine, CSF-1 and CCL2 in the context of macrophage activation, suggesting a spectrum of macrophage activation states. Future single-cell analysis of tumours will provide further insight into the complexity of macrophage activation, which will have ramifications for both veterinary and human cancer treatment programs.

## Methods

### Cell culture

RAW264.7 mouse macrophages, REM134 canine mammary cancer cell line [10], MCF7 human mammary cancer cell line, HEK293T human embryonic kidney and Lilly canine inflammatory mammary cancer cell line were used. All cell lines were grown in Dulbecco’s modified Eagle’s medium (DMEM) (Gibco) supplemented with 10 % FBS, penicillin-streptomycin (100 μg/mL) and glutamate (2 mM) (Gibco) in an atmosphere of 5 % CO_2_ and 95 % humidified air at 37 °C.

### Co-cultures

For flow cytometry assays, macrophage and REM134 cells were grown in co-cultures or in the presence of conditioned medium (CM) from RAW264.7 cells. For co-cultures, RAW264.7 macrophages were stained with CFSE (eBioscience). Cells were grown at a ratio of 2:1 of REM:RAW. Conditioned media was collected from sub-confluent cultures grown for at least 48 h and added at between 10–20 % of final culture volume. Co-cultures, cell cultures with conditioned medium or cells that received CSF1/CCL2/CCR2i were grown for at least 3 days, when LPS (Sigma, L4391) would be added at 1 μg/ml (when stated in the text) for at least 48 h. Cells were removed from plate by trypsinization or scraping.

### Drug treatment

RAW264.7 cells were treated with hrCSF-1 (Invitrogen) [50 ng/ml], CCL2 [50 ng/ml] (R&D Systems), canine recombinant CSF-1R [1 μg/ml] every 24 h for 48 h, unless indicated. Canine CSF-1R was expressed in HEK293T cells from a pEF6-V5-His vector (Invitrogen) containing the extracellular portion of the CSF-1R receptor. The receptor was affinity purified using Ni-NTA resin (Qiagen) before adding to cell culture.

RAW264.7, REM134 and Lilly cells were treated with CCR2 small molecule inhibitor (RS102895 hydrochloride - Sigma) [10 μM] and CSF1R inhibitor (GW2580) 48 h prior to analyses. DMSO was used as a vehicle control. For the effect of CCL2 on LPS activation of RAW264.7 cells, cells were incubated with CCL2 for 5 days before the addition of LPS.

### Enzyme-Linked Immunosorbent Assay (ELISA)

Determination of binding of CSF-1 to canine recombinant CSF-1R was performed by ELISA. Wells were coated with 500 ng of the extracellular portion of canine CSF-1R in 50 μl of PBS at 4 °C, overnight. Wells were blocked with 200 μl of PBS + 1 % BSA for 1 h at 37 °C. Human recombinant CSF-1 (Biolegend) was added at the stated concentrations. Plate was washed 6 x with PBS + 0.05 % Tween-20. Binding of CSF-1 was detected using anti-CSF-1 (Peprotech) at 1:200. A HRP-conjugated secondary swine anti-rabbit antibody was added at 1:1700 (Dako). The reaction was developed using TMBUltra® (Pierce). Colour development was stopped using 2 M H_2_SO_4_ and the plate was read at 450 nm.

### Cell viability

For cell proliferation assays, cells were grown in opaque 96-well plates. Conditions were tested on at least 4 replicates. GW2580, toceranib phosphate or RS102895 hydrochloride were added at the stated concentrations. Macrophage conditioned media was added to REM134 cells at 20 % of culture media volume. Cell proliferation was analysed with CellTiter-Glo® (Promega) following manufacturer’s instructions.

### siRNA mediated gene silencing

For transient transfection of small interfering RNA (siRNA) knockdown experiments, RAW264.7 cells were transfected with siRNA using the Amaxa Cell Line Nucleofector Kit V (Lonza,VCA-1003) according to the manufacturer's instructions. Commercially available TWIST1 and GAPDH siRNAs were purchased from Thermo Scientific - Accel SiRNA technology. Gene knockdown was validated by immunofluorescent staining of cells with an anti-Twist-1 antibody (see section below). LPS treatment and phagocytosis assays were carried out as described below.

### Flow cytometry

For analysis of receptor expression, after removal from culture plates, cells were incubated with the primary antibody for 30 min at 0 °C. Primary antibodies: anti-mouse MHCII I-A/I-E (eBioscience); anti-mouse CD301 (AbD Serotec). Phagocytosis analysis was performed using 2 μm latex beads (Sigma). The beads were opsonized for 30 min at 37 °C with mouse serum IgG (Sigma) before adding to the cells and incubating for 30 min at 37 °C. Glucose uptake was assessed using 2NBDG fluorescent glucose (Invitrogen) [50 μM] for 30 min at 37 °C or 0 °C. Samples were analysed on a FACScalibur (BD). Granularity was detected in the SSC channel, and fluorescence was detected using an argon laser. Sorting of RAW264.7 cells for determination of cell doublets was performed using a FACSAria II.

### Western blotting

For Western blots, cell lysates were obtained of confluent culture (lysis buffer: 7 M urea; 0.1 M DTT; 0.05 % Triton X-100; 25 mM NaCl; 20 mM Hepes-KOH, pH7.6). Equal amounts of protein were separated by SDS polyacrylamide gel electrophoresis (SDS-PAGE), transferred to Hybond-C nitrocellulose membrane (Amersham Pharmacia), and hybridised to an appropriate primary antibody and HRP-conjugated secondary antibody for subsequent detection by ECL. Primary antibodies used were rabbit anti-Twist1 (Santa Cruz Biotech); goat anti-COX-2 (Santa Cruz Biotech), mouse anti-β-actin (Abcam). Secondary antibodies anti-mouse, anti-goat and anti-rabbit were HRP-conjugated and were used following manufacturer’s recommendations (Dako).

### Immunofluorescence

For immunefluorescent staining, cells were grown on tissue culture treated chamber slides (Nunc Labtech) overnight. Cells were fixed with ice cold acetone for 20 min and blocked with 10 % goat serum. Rabbit anti-CSF1R (Abcam), rabbit anti-Twist1 (Sigma) were used. Anti-mouse or anti-rabbit Alexa conjugated antibodies (Invitrogen) were used as secondary antibodies at 1:200 in 10 % goat serum/PBS. Slides were mounted with aqueous mountant containing DAPI (Vectashield).

### Quantitative real time PCR

Total cellular RNA was extracted using RNeasy® (Qiagen). RNA quality was determined by A_260_ measurement. RT-PCR was performed with Omniscript (Qiagen) using random nonamer primers (Sigma). Quantitative RT-PCR (qPCR) was performed using Platinum SYBR (Invitrogen). Assay was run on Mx3000P (Stratagene). Primers are shown on Additional file 1.

### Sequencing of CSF-1R from a canine cancer cell line

mRNA was extracted from the Lilly inflammatory canine mammary carcinoma cell line using the RNeasy® kit (Qiagen). cDNA was synthesized using random nonamers (Sigma) and the Omniscript® (Qiagen) reverse-transcriptase following the manufacturer’s instructions. The receptor was amplified using the primers shown on Additional file 2 using Phusion High-fidelity polymerase (Thermo). The product was cloned into the pCR4-TOPO vector (Invitrogen) and sequenced using the T3 and T7 primers, and also the primers shown on Additional file 2. Sequencing performed at DNA Sequencing and Services, Dundee, Scotland.

### Statistical analysis

Normality tests were performed using the Minitab 16 software. Kruskal-Wallis, Student t-test and ANOVA statistical analyses (*P* < 0.05, unless indicated) were performed using GraphPad Prism 5. qPCR data was normalized to the control group and Log_2_ transformed before analysis. Unless indicated, all graphs are shown as mean ± standard deviation (SD).

## Additional files

Additional file 1:
**Primers for quantitative real time PCR.** Primers shown from 5’ end to 3’ end. Additional file 2:
**Primers for sequencing of CSF-1R from a cancer cell line.** Primers shown from 5’ end to 3’ end.

## Abbreviations

(p)STAT-3: (phosphorylated) signal transducer and activator of transcription 3
Ang1/2: angiopoietin 1/2
CCL2: Chemokine (C-C motif) ligand 2
CCR2: Chemokine (C-C motif) ligand 2 receptor
CCR2i: small-molecule inhibitor of the CCR2, RS102895 hydrochloride
CD: cluster of differentiation
cKit: Mast/stem cell growth factor receptor
CM: conditioned media
COX-2: cyclooxygenase
cr: canine recombinant
CSF-1: colony-stimulating factor 1 or macrophage colony stimulating factor
CSF-1R: colony stimulating factor 1 receptor
DMEM: Dulbecco's modified Eagle's medium
EGF: epidermal growth factor
ELISA: enzyme-linked immunosorbent assay
FBS: fetal bovine serum
hr: human recombinant
HRP: horseradish peroxidase
IFNγ: interferon gamma
IL: interleukin
LPS: lipopolysaccharide
MHC: main histocompatibility complex
NS: not significant
PCR: polymerase chain reaction
SD: standard deviation
siRNA: small interfering RNA
TAMs: tumour-associated macrophages
TLR: Toll-like receptor
TNFα: tumour necrosis factor α
VC: vehicle control
VEGF: vascular endothelial growth factor
