# Supplementary material for: Canine mammary cancer cells direct macrophages toward an intermediate activation state between M1/M2
Source: BMC Vet Res. 2015 Jul 15;11:151. doi: 10.1186/s12917-015-0473-y (PMC4502937; doi:10.1186/s12917-015-0473-y)
Supplement: Additional file 2: — Primers for sequencing of CSF-1R from a cancer cell line. Primers shown from 5’ end to 3’ end. [file 12917_2015_473_MOESM2_ESM.docx]

| Canine full CSF-1R receptor cloning | Forward: ATGGGCCTAGGGGCTCCACTG  Reverse: GCAGAACTGGTAGTTGTTGGGCTGCAGCAG |
| --- | --- |
| Sequencing primers | CCGAGTACTGTTGCTATGGTGAC  CTTCCACAAGGTGATCGTCCAC  GATGATCTTCCAGCGCACCT |
